# Characterization, cytotoxicity, and genotoxicity properties of novel biomediated nanosized-silver by Egyptian *Streptomyces roseolus* for safe antimicrobial applications

**DOI:** 10.1007/s11274-022-03231-6

**Published:** 2022-01-27

**Authors:** Asmaa Elnady, Noha M. Sorour, Rateb N. Abbas

**Affiliations:** 1 Department of Microbial Biotechnology, Genetic Engineering and Biotechnology Research Institute (GEBRI), University of Sadat City, Sadat City, 22857/79, Egypt; 2 Department of Industrial Biotechnology, Genetic Engineering and Biotechnology Research Institute, University of Sadat City, Sadat City, 22857/79, Egypt

**Keywords:** *Streptomyces*, Antimicrobial, Cytotoxicity, Genotoxicity, NanoSilver

## Abstract

Multi-drug resistant bacteria are not effectively managed with current treatments, making it a serious global problem. Therefore, there is an essential need for finding new antimicrobial agents. In this regard, silver nanoparticles (Ag-NPs) have been projected as a new generation of antimicrobial agents. Ag-NPs were biomediated by Egyptian *Streptomyces roseolus* for the first time, which was molecularly identified using 16S rRNA sequencing under accession no. MT071505. Biosynthesized Ag-NPs were characterized using UV–Vis spectroscopy, XRD, TEM, FTIR, and DLS. FTIR analysis confirmed the presence of different bioactive functional groups, such as N–H, C–H, C–O–C, C–NH_2_, and C=O acting as bioreducing/stabilizing agents for Ag-NPs. Ag-NPs exhibited antimicrobial activity against some multi-drug resistant Gram-positive and Gram-negative pathogens. MBC of Ag-NPs against *Listeria monocytogenes* and *Klebsiella pneumonia* were 0.195 and 0.048 mg/mL, respectively, with a tolerance level of 2 confirming its biocidal effect. SEM imaging of Ag-NPs-treated *L. monocytogenes* and *K. pneumonia* showed shrunk destroyed cells after 6 h of treatment. Biosynthesized Ag-NPs exhibited IC_50_ of < 0.3 and 8.21 mg/mL, on normal Human Skin Fibroblast, and Blood Lymphocytes, respectively. IC_50_ values were significantly higher than its MBC values, with no harmful cytotoxic effect, thus can be safely applied at its biocidal concentration. For biosafety purposes, the genotoxicity of biosynthesized Ag-NPs was assessed using Comet assay for the first time on Blood Lymphocytes, with zero-tail and 100% head intensity indicating non-genotoxic effect. An ecofriendly biomediated synthesis of Ag-NPs was described with easy scale-up, non-toxic by-products, so, it can be recommended as a powerful-safe antimicrobial agent.

**Graphical abstract:**

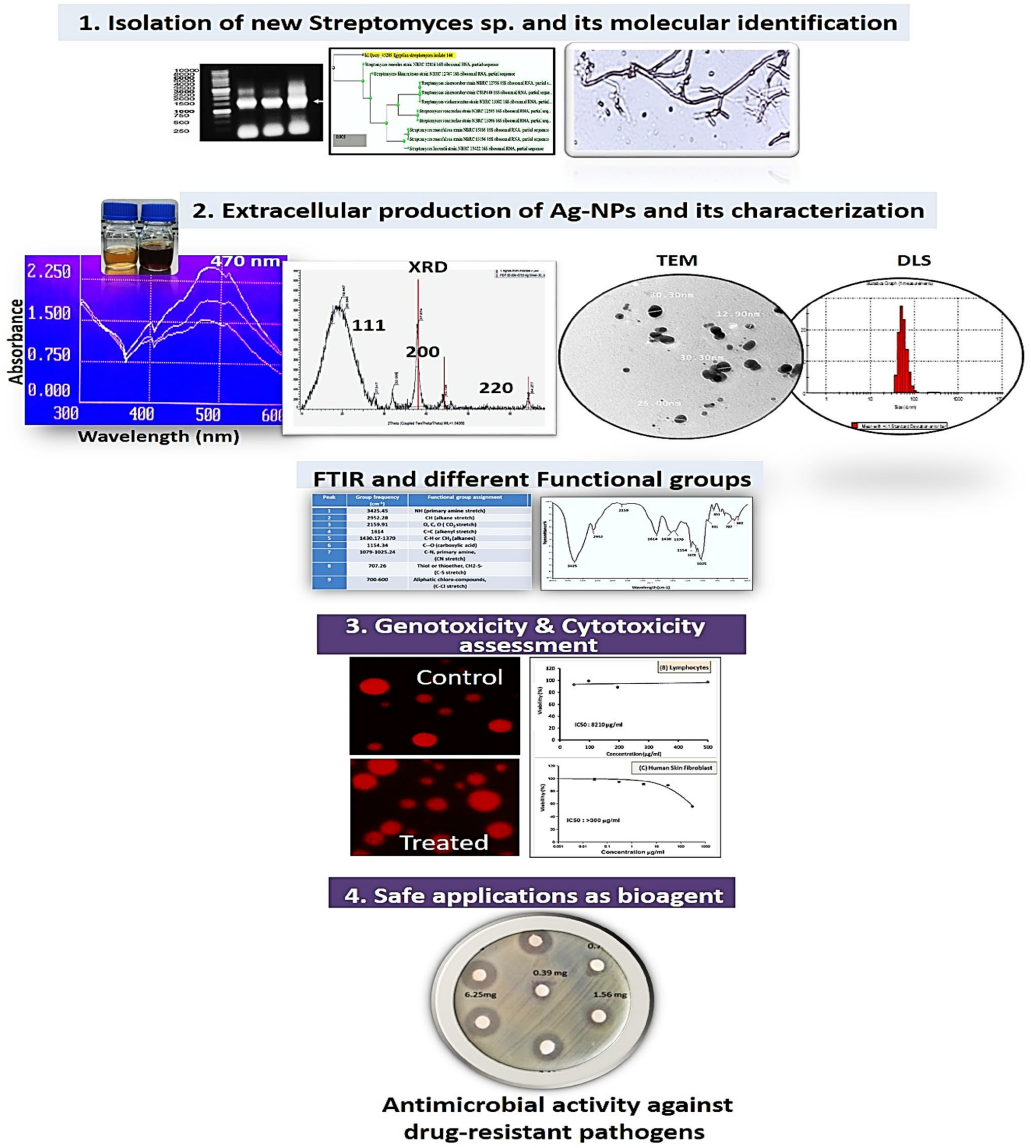

## Introduction

Multi-drug resistant bacteria are not effectively managed with current treatments, making it a serious global problem for public health. Accordingly, there is a strong need to develop new bactericides (Wypij et al. 2018). The increasing incidence of multidrug-resistant pathogens responsible for nosocomial infections has been detected in different health-care facilities in Egypt, where, Gram-negative bacteria were the most common pathogens (Saied et al. 2011; Fakhr and Fathy 2018; Hassan et al. 2021). The antibiotic resistance pattern among patients showed the prevalence of methicillin-resistant *S. aureus* (MRSA), extended-spectrum beta-lactamase bacteria, Cephalosporin resistant *K. pneumoniae*, Carbapenem resistant *K. pneumonia*, and the multi-drug resistant-*P. aeruginosa*, *E. coli*, and *Acinetobacter baumannii* spp. (Fakhr and Fathy 2018; Hassan et al. 2021).

Recently, metallic nanoparticles (NPs) have gained great significance in the modern era of nanomaterials because of their unique intrinsic properties based on their morphology, size, and distribution (El‐Baz et al. 2016; Mourdikoudis et al. 2018). These new properties are excellent physicochemical, electrical conductivity, catalytic, and mechanical stability (Srikar et al. 2016; Hassan et al. 2019; Crisan et al. 2021). Among metallic NPs, Ag-NPs have found tremendous applications in catalysis, biosensing, drug delivery, and medicine, etc. Currently, there has been an increasing interest in Ag-NPs because of their antimicrobial property, and even has been projected as a new generation of antimicrobial agents (Martínez et al. 2021). Therefore, Ag-NPs have been exploited for various applications, in household and healthcare-related products, wound dressings, medical device coatings, and cosmetics (Zhang et al. 2016; Wypij et al. 2018; Vanlalveni et al. 2021).

Although physicochemical synthesis generates stable monodispersed NPs with various shapes, these methods aren’t recommended, because they involve the use of chemical reagents, require costly reaction setup, and generate toxic end-products causing environmental pollution (Srikar et al. 2016; Yaqoob et al. 2020). These drawbacks, make it very important to search for eco-friendly, and cost-effective approaches for the synthesis of NPs. Interestingly, microorganisms are considered nano-factories that play a vital role in the bioremediation of toxic metals through the reduction of metal ions (Anandaradje et al. 2020). In this respect, the actinomycetes members of the bacterial order Actinomycetales are found worldwide in soil, where *Streptomyces*, the largest genus of actinobacteria are considered efficient candidates for extracellular biosynthesis of metallic NPs with very good stability (Wypij et al. 2018). Furthermore, microorganisms or their secondary metabolites contain different biomolecules, which act as reducing and/or stabilizing agents for Ag-NPs and represent a safe approach than the physicochemical methods (Anandaradje et al. 2020). These natural reducing agents contain many functional groups which can increase the reduction capability and improve the stability of Ag-NPs (Zhang et al. 2016). Herein, the main objective of this study was to achieve a green approach for the biosynthesis of Ag-NPs using new *Streptomyces* strain isolated from the Egyptian soil. Characterization of the produced Ag-NPs was investigated, and its antimicrobial activity was tested against different pathogens. For biosafety applications, the cytotoxicity effect of biosynthesized Ag-NPs has been tested on different cell lines, and for the first time, its genotoxicity was assessed using Comet assay.

## Material and methods

### Materials

AgNO_3_ as a precursor for Ag-NPs synthesis was obtained from Sigma/Aldrich, USA. Peptone, Yeast extract, Malt extract, Glucose, Starch, and Dimethyl Sulphoxide (DMSO) were purchased from Lobal Chemie, India. All materials used for cell culture experiments were obtained from Cambrex BioScience (Copenhagen, Denmark). All other chemicals and reagents were bought locally and were analytically reagent grade from Algomhoryia Company for Chemicals, Cairo, Egypt.

### Culture media and microorganisms

Glucose Yeast Malt medium was used for actinomycetes isolation having the following composition (g/L); malt extract 10, yeast extract 3, glucose 3, agar 20, and pH 7.3 ± 0.1. Starch nitrate medium was used as a synthetic medium for the biosynthesis of Ag-NPs (Abd-Elnaby et al. 2016) with the following composition (g/L); starch 20, K_2_HPO_4_ 0.5, KNO_3_ 1, MgSO_4_.7H_2_O 0.5, FeSO_4_ 0.01, KCl 0.5, and pH 7.2 ± 0.2. Nutrient agar medium was used for growth and maintenance of pathogens with the following composition (g/L); beef extract 3, peptone 5, NaCl 5, agar 20, and pH 7. Gram**-**positive bacteria (*Bacillus cereus* ATCC 14,579, *Bacillus subtilis* ATCC 6633*, Listeria monocytogenes* ATCC 19,116*, Staphylococcus aureus* ATCC 6538), Gram-negative strains (*Escherichia coli* O157:H7*, Klebsiella pneumonia, Aeromonas hydrophilia*), and Yeast (*Candida albicans*) were obtained from Dar Al-Fouad Hospital at 6th of October City, Cairo, and El-Mabarra Educational Hospital, Alexandria, Egypt.

### Isolation of actinomycetes

Soil samples were collected in sterile plastic bags from Menuf, Minufyia governorate, Egypt. 10 g of collected sample was suspended in 90 mL of saline solution (0.9% NaCl, w/v), serially diluted, and 1 mL aliquot of each dilution was plated on sterile agar plates containing Glucose Yeast Malt Agar medium and was incubated for 5 days at 30 °C. Colonies that were morphologically identified as actinomycetes were selected. Pure developed single colonies were obtained by conventional streak plate technique, kept at 4 °C on slants. All cultures were routinely stored in glycerol solution (25% v/v) at − 80 °C.

### Biosynthesis of Ag-NPs

Cell-free extract of the isolated actinomycete was grown in 250 mL-Erlenmeyer flask containing starch nitrate broth medium, incubated in a rotary shaker (New Brunswick, CA) at 170 rpm and 30 °C for 3 days. After the incubation, culture was centrifuged at 6000 rpm for 20 min, and the collected supernatant was used for the biosynthesis of Ag-NPs. 50 mL of AgNO_3_ solution (1 mM) was mixed with 50 mL of the actinomycete supernatant (Abd-Elnaby et al. 2016). The mixture was incubated at 30 °C and 170 rpm agitation at different temperature degrees (30, 45, and 60 °C) for 24 h at 150 rpm in the rotatory shaker. Control experiments were carried out by mixing AgNO_3_ solution and un-inoculated media or adding the actinomycete supernatant without AgNO_3_. The reduction of Ag^+^ ions was checked by the color change of the reaction mixture.

## Characterization of biosynthesized Ag-NPs

### UV–Visible spectroscopy (UV–Vis) and X-ray diffraction (XRD)

The formation of Ag-NPs during the biosynthesis was monitored using a UV–Vis spectrophotometer (Shimadzu T80 spectrophotometer, China). 1 mL aliquot of biosynthesized Ag-NPs colloidal solution was scanned at a wavelength ranging from 300 to 700 nm. The crystalline natures of the biosynthesized Ag-NPs powder were investigated by X-ray diffraction (Bruker D2 Phaser diffractometer 2nd Gen.), operating at (50 kV and 0.5 mA), with a Cu anode radiation (1.54060 Å) in the angular range of 10°-70° using continuous scanning 2*ϴ* mode. The size of the Ag-NPs formed in the bio-reduction process was determined using Scherrer's formula (Sumadevi et al. 2021).

$${\text{D}} = {\text{K}}\lambda /\beta {\text{ cos}}\theta$$

K is the Scherrer constant (shape factor), λ is the X-ray wavelength (1.5418 Å), β is the width of the XRD peak at half-height, θ is the Bragg angle, and D is the grain size.

### Transmission electron microscope (TEM), dynamic light scattering (DLS), and fourier transform infrared (FTIR)

The size and morphology of the biosynthesized Ag-NPs were studied by TEM (FETEM, JSM-2100F, JEOL Inc.) at Petroleum Research Institute, Cairo, Egypt. An aliquot of Ag-NPs suspension was transferred onto carbon-coated copper grid, and allowed to dry. The grid was then scanned using TEM (Phillips EM 208S) operated at 100 kV voltage. Ag-NPs were analyzed using Nano-Zeta Sizer (Nano ZS, ZEN 3600, Malvern Nano, UK) to determine the distribution of particles size. DLS measures the light scattered from a laser when passes through the sample and the fluctuations of the scattered light are detected at a known scattering angle θ by a fast photon detector (Fissan et al. 2014). The FTIR spectrum of the dried biosynthesized Ag-NPs sample was recorded on an FTIR spectrometer 8000 series, with KBr in the wavenumber region of 4000–400 cm^−1^ at a resolution of 4 cm^−1^. To identify the functional groups found in the tested sample, the spectral data recorded were compared with the reference database.

### Molecular identification and phylogenetic analysis

The selected actinomycetes isolate was identified using 16S rRNA gene partial sequencing method (Kim et al. 2011). Genomic DNA of the selected actinomycetes isolate was extracted using genomic DNA Extraction Kit (Intron, Biotechnology, Korea). PCR amplification of 16S ribosomal DNA (16S rDNA) was performed with a set of universal primers; 27F-Forward primer 5′AGA GTT TGA TCC TGG CTC AG 3′ (20 mer) and 1492R-Reverse primer 5′CTA CGG CTA CCT TGT TAC GA 3′ (20 mer). The PCR amplification was performed using a programmed thermal cycler (Thermo Fisher Scientific, USA). The produced amplicons of the selected isolate (PCR product) were tested for their quality by electrophoresis in Bio-Rad submarine (8 × 12 cm) using agarose gel (1%) and compared with 1 kb DNA ladder (Intron Biotechnology, Korea). DNA banding patterns of 16S gene amplicons were visualized using a UV-transilluminator (Thermo Fisher Scientific, USA) under UV light. PCR products were purified using gene JET™ genomic DNA purification kit (Intron Biotechnology, Korea) according to the manufacturer's instructions. Then sequenced using forward and reverse primers with ABI 3730xl DNA sequencer. Nucleotide bases obtained after sequencing were identified and compared with similar sequences retrieved from the GenBank database within the National Center for Biotechnology Information (NCBI) (http://www.ncbi.nlm.nih.gov/GenBank/index.html) using nucleotide Basic Local Alignment Search Tool (BLAST) Gene Sequences in the database website (http://www.ncbi.nlm.gov/BLAST/). The nucleotide sequences of the 16S rRNA genes was deposited under accession number MT071505. The phylogenetic analysis of sequences was created using MEGA integrated software for Molecular Evolutionary Genetics Analysis and sequence alignment (http://www.megasoftware.net/). The statistical method is maximum likelihood, and the test of phylogeny is Bootstrap method with no. of bootstrap replication equal to 500 based on Tamura-Nei model (Tamura and Nei 1993; Stecher et al. 2020).

## Qualitative and quantitative assessment of antimicrobial activity

### Bacterial strains and growth conditions

The antimicrobial activity of the biosynthesized Ag-NPs was tested against different Gram-positive bacteria (*L. monocytogenes*, *S. aureus*, *B. subtilis*, *B. cereus*), Gram-negative bacteria (*E. coli* O157:H7, *K. pneumonia*, *A. hydrophilia*), and yeast (*C. albicans*). All bacterial strains were grown on nutrient broth, except *L. monocytogenes* was grown on brain heart infusion (Oxoid, ltd, England), and the yeast was grown on Wickerham medium. Stock inoculum suspensions of the pathogenic strains were freshly prepared by picking colonies from 24 h cultures grown on at 37 °C and suspended in sterile saline solution (0.9% NaCl, w/v). The optical density of pathogens was adjusted to achieve turbidity equivalent to 0.5 McFarland standard, approximately (1 × 10^8^ CFU/mL) for bacteria, and (1 × 10^6^ CFU/mL) for *C. albicans* (Andrews 2001).

### Disc diffusion method

Disc diffusion method is commonly used as a preliminary screening test prior to quantitative minimal inhibitory concentration (MIC) determination. Microbial inocula were spread on plates containing appropriate medium using sterile cotton swab, and plates were allowed to dry for 15 min at room temperature. Sterile filter paper discs (Hi media) were saturated by 40 μL of two-fold serially diluted concentrations of biosynthesized Ag-NPs in the range (0.39–25 mg/mL). Discs saturated with the actinomycete supernatant and AgNO_3_ were used as a negative and positive control, respectively. The plates were incubated at 4 °C for 1 h to allow the diffusion of Ag-NPs into the medium and were incubated at 37 °C for 24 h (Wikler 2006). After incubation, inhibition zones diameters were measured in mm, and MIC was determined as the lowest concentration of Ag-NPs that produced an inhibition zone after 24 h of incubation.

### Agar dilution method

Brain Heart Infusion (BHI) and nutrient agar (NA) media were used for the growth of *L. monocytogenes* and *K. pneumonia*, respectively. Two-fold serial dilutions of biosynthesized Ag-NPs were prepared in molten BHI agar and NA medium with desired final concentrations. Using the pour-plate method (Klančnik et al. 2010), 100 μL of *L. monocytogenes* or *K. pneumonia* bacterial suspensions (0.5 Mcfarland ~ 1:2 × 10^8^ CFU/mL) were inoculated into appropriate agar medium and incubated at 37 °C for 24 h. An inoculated agar plate without Ag-NPs was served as a positive control and another one without inoculum as a negative control.

### Broth macro-dilution method, MIC, and MBC determination

Broth macro-dilution was done according to Andrews (2001) method. The biosynthesized Ag-NPs were added to 10 mL of BHI broth medium and nutrient broth to give final concentrations, with respect to the results obtained by the agar dilution method. 100 μL from the diluted culture of *L. monocytogenes* or *K. pneumonia* (0.5 ~ Mcfarland 1:2 × 10^8^ CFU/mL) were inoculated in growth media containing the desired concentration of Ag-NPs, well shaken, and incubated at 37 °C for 24 h. Bacterial growth was followed by plating 1 mL from the incubated culture tubes and control on suitable media. After 24 h of incubation, plates were compared with the positive control, and the bacterial colony number was calculated as (CFU/mL). The MIC is the lowest concentration of biosynthesized Ag-NPs resulting in a significant reduction (99%) of pathogen viability after 24 h of incubation. The concentration where 100% of microbial growth was inhibited as compared to the negative control (media only) was designed as MBC value. The tolerance level of pathogen towards the biosynthesized Ag-NPs was calculated using the following formula (May et al. 1998):

$${\text{Level}}\,{\text{of}}\,\,{\text{Tolerance}} = {\text{MBC}}/{\text{MIC}}$$

### Scanning electron microscope (SEM) imaging

The effect of biosynthesized Ag-NPs on pathogens was studied using SEM, micrographs were captured using Jeol JSM- 5300 SEM operated between 15 and 20 keV at Faculty of Science, Alexandria University, Egypt. Samples were prepared according to Tamboli and Lee (2013) method with some modifications as follows; 10 mL of fresh bacterial culture of *L. monocytogenes* and *K. pneumonia* grown in Luria–Bertani medium for 18 h were treated with biosynthesized Ag-NPs at their MIC, then incubated for 3 and 6 h. Treated bacterial cells were collected by centrifuging at 6000 rpm for 10 min. Culture supernatants were discarded and the treated cells were washed by phosphate saline buffer (pH 7.4) to remove excess of Ag-NPs, then centrifuged to collect washed cells. Samples were fixed by immersing immediately in an equal volume of a fixative solution (1% glutaraldehyde, 4% paraformaldehyde in 0.1 M sodium-cacodylate) at 4 °C for 24 h. Samples were then post-fixed in 2% OsO_4_ in the phosphate saline buffer (pH 7.4) at 4 °C for 2 h. Samples were washed in the buffer and dehydrated at 4 °C through graded series of ethanol then dried by N_2_ gas. Finally, the samples were mounted using carbon paste on AL-stub using double-sided conductive tapes and coated with gold up to a thickness of 400 A in a sputter-coating unit (JFC-1100 E).

### Cytotoxic assessment of biosynthesized Ag-NPs

Cell viability and cytotoxicity of biosynthesized Ag-NPs were tested on normal Human Skin Fibroblast (HSF) cell lines using sulforhodamine B protein (SRB) assay and on Peripheral Blood Lymphocytes normal cells, as well as Human Hepatocellular Carcinoma (HepG-2) cell lines using MTT assay. Normal HSF cell lines were obtained from Nawah Scientific Inc., (Mokatam, Cairo, Egypt). Cells were maintained in Dulbecco’s Minimum Essential Medium (DMEM) supplemented with 100 mg/mL of streptomycin, 100 U/mL of penicillin, and 10% of heat-inactivated fetal bovine serum in humidified CO_2_ (5% v/v) at 37 °C. Aliquots of 100 μL cell suspension (5 × 10^3^ cells) were distributed in 96-well plates and incubated for 24 h. Cells were treated with an aliquot of 100 μL media containing Ag-NPs (0–300 μg/mL) concentrations. After 72 h of exposure, cells were fixed using 150 μL of TCA (10%) and incubated for 1 h at 4 °C. The TCA solution was removed, and cells were washed five times using double distilled water. Aliquots of 70 μL SRB solution (0.4% w/v) were added and incubated at 25 °C in darkness for 10 min. Plates were washed three times with acetic acid (1% v/v) and allowed to air-dry. To dissolve the protein-bound SRB stain, 150 μL of TRIS (10 mM) was added, and the absorbance was measured at 540 nm using a BMGLABTECH®-FLUO star Omega microplate reader, Ortenberg, Germany (Skehan et al. 1990).

Hep-G2 cell lines (ATCC, USA) were used to evaluate the cytotoxic effect of the biosynthesized Ag-NPs using 3-[4,5-dimethylthiazole-2-yl]-2,5-diphenyltetrazolium bromide (MTT) assay. Cells were routinely cultured in DMEM, supplemented with 10% FBS, 2 mM L-glutamine, 100 U/mL streptomycin-sulfate, 250 ng/mL amphotericin B, and 100 U/mL sodium penicillin-G. Cells were maintained in humidified air containing 5% CO_2_ at 37 °C. For sub-culturing, the monolayer cells were harvested after trypsin/EDTA treatment at 37 °C. Peripheral Blood Lymphocytes were isolated from fresh blood samples according to Kizhakeyil et al. (2019). Lymphocyte cell suspension was centrifuged at 2000 rpm for 10 min, washed twice, then suspended in incomplete RPMI-1640. Lymphocyte cell viability was checked using Trypan blue stain (Sigma). The cells (2.5 × 10^5^ cells/mL) were cultured in RPMI-1640 supplemented with 10% FBS as triplicates of 200 µL/well into flat-bottom 96-well tissue culture plates (Griener) and incubated with biosynthesized Ag-NPs (48–500 µg/mL) for 48 h in a humidified 5% CO_2_ atmosphere. HepG-2 cells (0.5 × 10^5^ cells/well), in serum-free medium, were plated in a flat-bottom 96-well microplate, and treated with 20 µL of different Ag-NPs concentrations in the range (62.5–500 µg/mL) for 24 h at 37 °C, in a humidified 5% CO_2_. After incubation, 40 µL of MTT solution/well were added after the removal of media and incubated for an additional 4 h. MTT crystals were solubilized by the addition of 180 µL of DMSO/well, and plates were shacked at room temperature, followed by photometric determination of the absorbance at 570 nm using microplate ELISA reader (SunriseTM, Tecan Group Ltd. Männedorf/Switzerland). Each concentration was carried out in triplicate, the average and standard deviation were calculated. Data were expressed as the percentage (%) of relative viability as compared with untreated cells, with cytotoxicity indicated by < 100% relative viability. The extent of MTT reduction was quantified, where the number of viable cells was directly proportional to the intensity of formazan dark-blue color (Hansen et al. 1989). IC_50_ was calculated from the dose–response curve and the percentage (%) of relative viability was calculated using the following equation (Hansen et al. 1989):

$$[{\text{Absorbance}}\,{\text{of}}\,{\text{ treated}}\,{\text{ cells}}/{\text{Absorbance}}\,{\text{ of }}\,{\text{control}}\,{\text{ cells}})] \times {1}00$$

### In vitro genotoxicity alkaline Comet assay

The Comet assay was assessed according to Singh et al. (1988) with some modifications. Treated cells were incubated with different concentrations of biosynthesized Ag-NPs (48–500 µg/mL) for 12 h, under proper sterilized anaerobic conditions. Cells were washed by phosphate saline buffer (pH 7.4), resuspended in 0.75–1% low dissolving agarose (Bio-Rad), and were spread onto slides covered by 1% typical agarose. Slides were left to solidify at 40 °C for 5 min, then were placed in lysis buffer (2.5 M NaCl, 10 mM Tris, 100 mM EDTA, 1% TritonX100, 10% DMSO, pH 10) overnight at 40 °C. Slides were placed in unwinding buffer (300 mM NaOH, 1 mM EDTA, pH ˃ 13) for 40 min at 40 °C. Electrophoresis was carried out for 20 min at 25 V (1.0 V/cm), and 300 mA. Finally, the slides were neutralized using 0.4 M Tris buffer (pH 7.5) and were stained with ethidium bromide (20 µg/mL), then examined using a fluorescence light microscope (Leica DMi8 S-platform, Germany), 100 × objective lens. The tail intensity %, head intensity, and tail moment were assessed using Comet examine IV software.

### Statistical analysis

All experiments were conducted in triplicates, the mean and all standard deviations were calculated using Excel software (Microsoft office, 2010). Data were expressed in their mean values ± SD (standard deviation).

## Results

### Biosynthesis of Ag-NPs

**Fig. 1:**
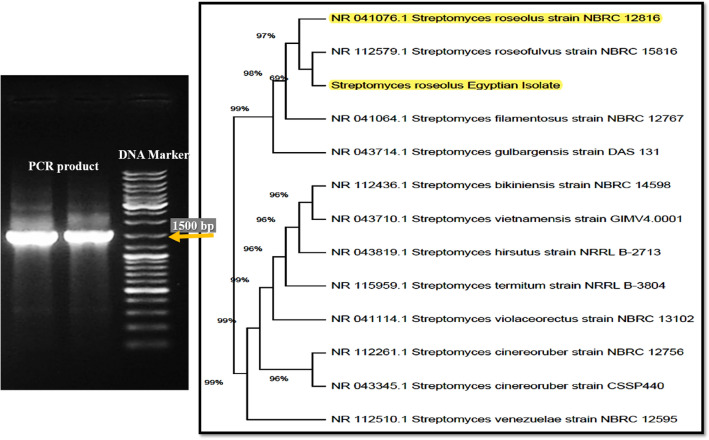
Phylogenetic tree of 16S sequences of the *Streptomyces* isolate #140 aligned with closely related sequences accessed from the GenBank under accession no. MT071505

**Fig. 2:**
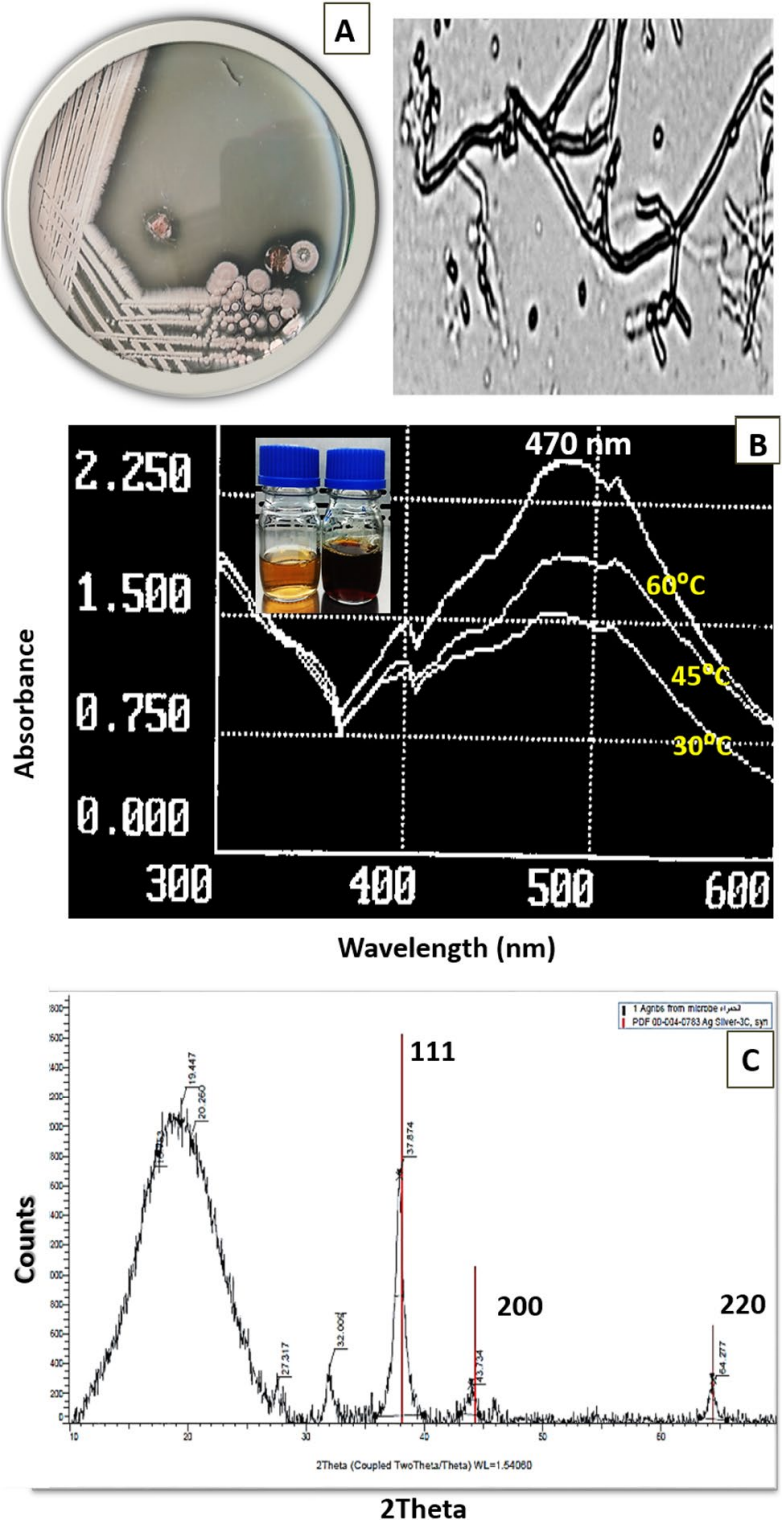
*Streptomyces roseolus* grown on starch nitrate agar medium and under light microscope *100 (**A**), UV–Vis Scanning of biosynthesized Ag-NPs by *S. roseolus* culture supernatant at different temperature (**B**), and XRD analysis of biosynthesized Ag-NPs (**C**)

In this study, Ag-NPs were successfully synthesized extracellularly using the culture supernatant of new *Streptomyces* strain isolated from the Egyptian soil (Fig. 1). Interestingly, the culture supernatant incubated with 1 mM AgNO_3_ mediated the biosynthesis of Ag-NPs within 6 h of incubation. The color of the reaction mixture was changed from yellow to dark reddish-brown, which indicates the formation of Ag-NPs (Fig. 2), whereas, no color change was observed in either the culture supernatant without AgNO_3_ or un-inoculated media with AgNO_3_ as control. After 24 h, the color was stable indicating that AgNO_3_ was completely reduced, and the reaction has come to an end.

### Molecular identification and the phylogenic tree of selected isolate

PCR amplification of the DNA samples from the selected *Streptomyces* isolate #140 generated a PCR product (Fig. 1) of expected size (1500 bp). The analysis of 16S rRNA gene of the *Streptomyces* isolate #140 was sequenced with 27F and 1492R primers at the forward and reverse directions. The NCBI database showed 98.74% of similarity with *Streptomyces roseolus*, under accession no. MT071505. Gene bank nucleotide database using the blast-n algorithm revealed significant matches with hi max score of 1818, zero e value, and 98.74% nucleotide identity for forward 16 s rRNA gene sequence of *Streptomyces* isolate #140 and that of *Streptomyces roseolus* strain (NBRC 12,816). Also, the reverse sequence shows hi max score of 1984 and zero e value with 98.01% identity with *S. roseolus* strain (NBRC 12,816)*.* The phylogenetic tree (Fig. 1) shows high genetic relationship between the Egyptian *Streptomyces* isolate #140 and *S. roseolus* strain (NBRC 12,816). The phylogenic tree shows a set of possible nucleotides at each ancestral node based on their incidental likelihood at site 1. Initial heuristic tree(s) were obtained using BioNJ algorithms and Neighbor-Join to a matrix of pairwise-distances using Tamura-Nei model. Topology was selected with superior log likelihood value and the analysis involved 13 nucleotide sequences (Fig. 1).

### Characterization of biosynthesized Ag-NPs

**Fig. 3:**
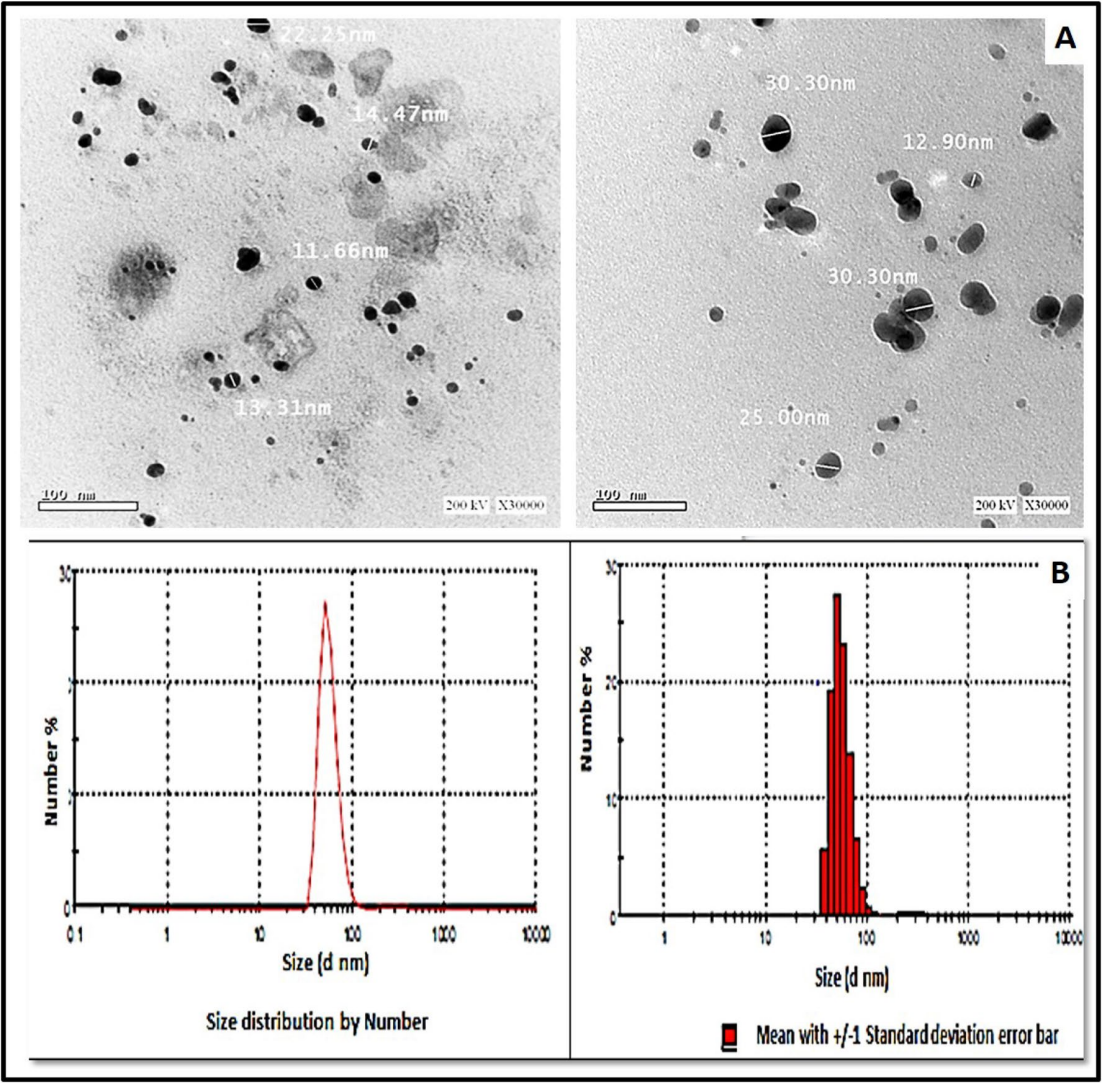
TEM analysis of Ag-NPs biosynthesized by *S. roseolus* culture supernatant, and hydro-dynamic size determination of biosynthesized Ag-NPs by *S. roseolus* using DLS

**Fig. 4:**
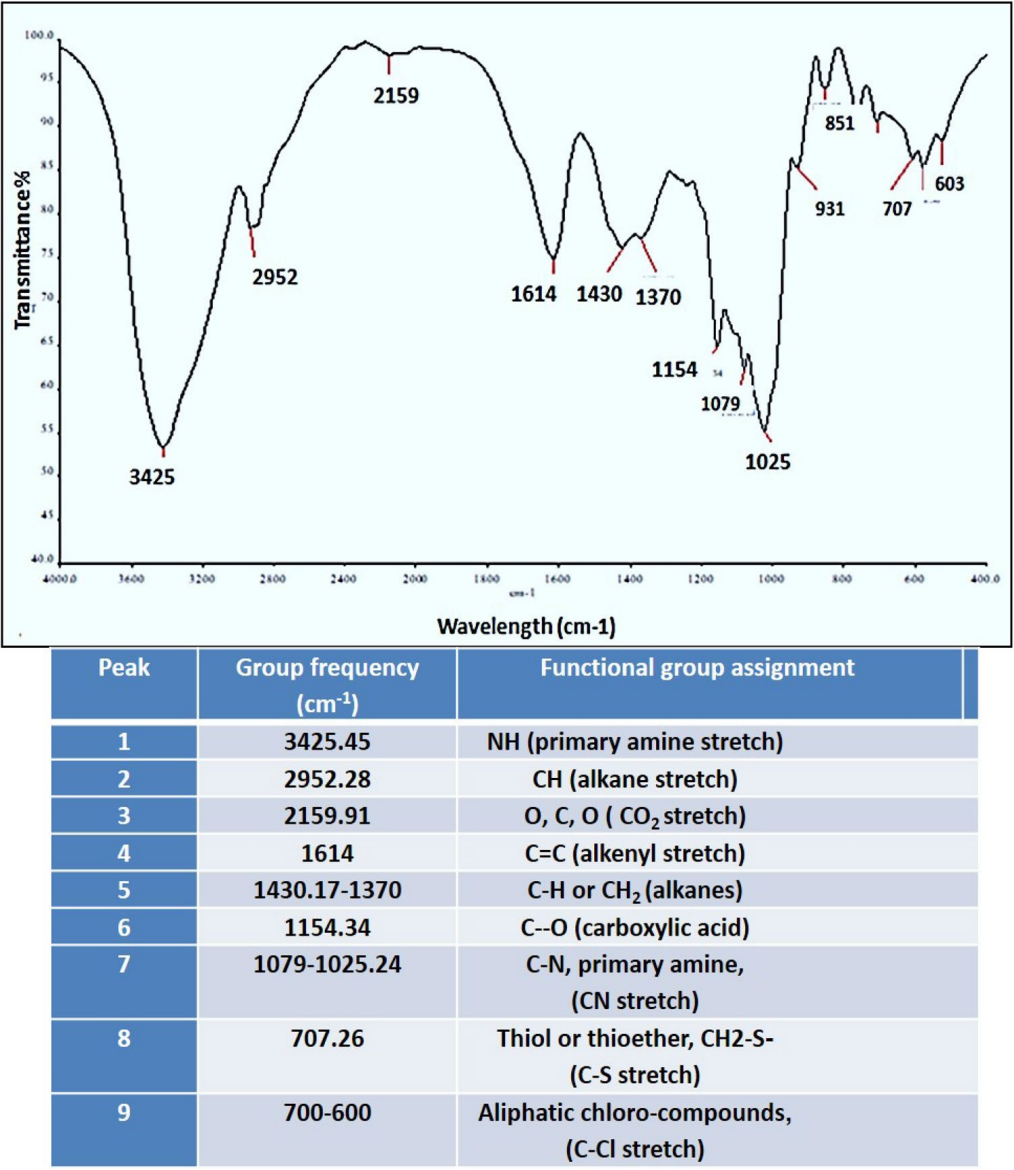
FTIR analysis of Ag-NPs biosynthesized by *Streptomyces roseolus,* showing different functional groups, such as, N–H, C-H, C–O–C, C-S, C-NH_2_, and C = O acting as bioreducing/stabilizing agents for the biosynthesized Ag-NPs

A strong and broad surface-plasmon-resonance (SPR) band maximum was observed at 470 nm, characteristic for Ag-NPs (Fig. 2). The color intensity was increased with increasing the temperature, and consequently, the intensity of SPR peaks was increased (Fig. 2). The XRD pattern of the biosynthesized Ag-NPs by *S. roseolus* (Fig. 2) showed characteristic XRD diffraction peaks centered at 37.874, 43.734, 64.277 induced by the crystalline planes of Ag as, 111, 200, and 220, respectively, as indexed planes of cubic face-centered silver. Peaks intensity reflects the high degree of Ag-NPs crystallinity. According to Scherrer's equation, the average size of Ag-NPs was found to be 10.8 nm. TEM micrograph showed the formation of well-dispersed spherical Ag-NPs biosynthesized by *S. roseolus* (Fig. 3). The size of Ag-NPs determined by TEM was 5–22 nm. The DLS pattern demonstrated that Ag-NPs biosynthesized by *S. roseolus* had a Z average diameter of 56.33 nm, and its poly-dispersity index (PDI) was 0.398. Results (Fig. 3) showed a bimodal scattered intensity distribution with a major maximum at about 56 nm. The FTIR analysis (Fig. 4) verified the presence of different functional groups on the surface of biosynthesized Ag-NPs. Results showed specific strong absorption bands at 3420, 2952, 1614, and 1430 cm^−1^. Different absorbance peaks like 1370, 1154, and 1062 cm^−1^ were also detected.

### Antimicrobial activity of Ag-NPs

**Fig. 5:**
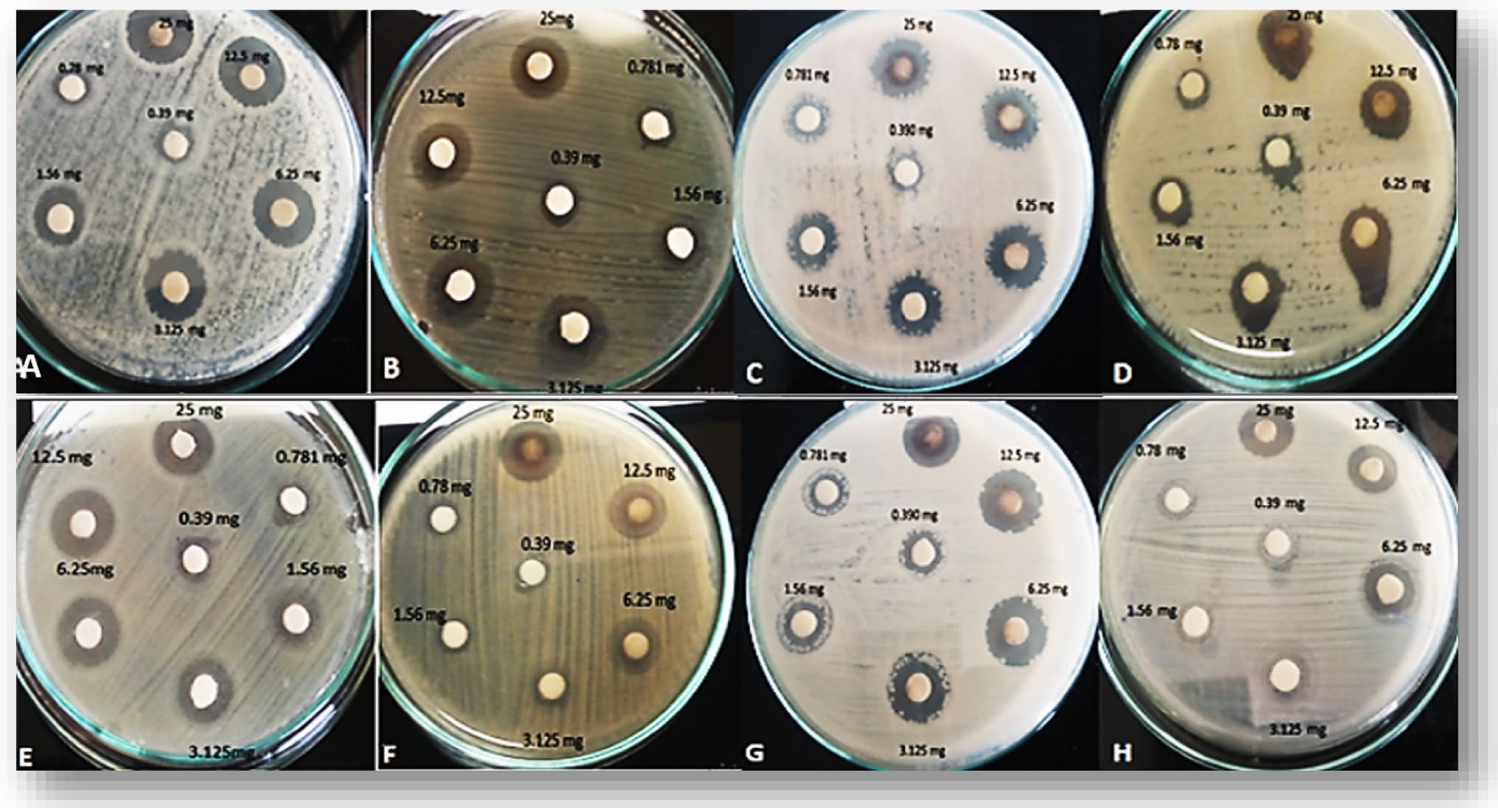
Antimicrobial activity of biosynthesized Ag-NPs was tested using disc diffusion method by two-fold dilution against *L. monocytogenes* (**A**), *S. aureus* (**B**), *B. subtilis* (**C**), *B. cereus* (**D**), *E. coli* O157:H7 (**E**), *A. hydrophilia* (**F**), *K. pneumonia* (**G**), and *C. albicans* (H)

The biosynthesized Ag-NPs exhibited an excellent dose-dependent antimicrobial activity against different Gram-negative, Gram-positive bacteria, and yeast (Fig. 5 and Table 1). The highest antimicrobial activity was against Gram-negative bacteria. Among all pathogenic strains, the biosynthesized Ag-NPs exhibited higher antibacterial activity against *E. coli* O157:H7 with inhibition zone diameter of 23.66 ± 0 mm (Table 1). The lowest activity was against *C. albicans* and *B. subtilis* with an inhibition zone of 18.66** ± **0.3 and 19.66** ± **0.3 mm, respectively.

**Table 1:** Antimicrobial activity of Ag-NPs biosynthesized by *S. roseolus* against different multidrug-resistant strains, measured as inhibition zone diameter in mm

Conc. (mg/mL)	Zone of Inhibition (mm)							
	Gram-positive strains				Gram-negative strains			Yeast
	*L. monocytogenes*	*B. cereus*	*B. subtilis*	*S. aureu*s	*Aeromonas hydrophilia*	*E. coli* O157:H7*	*K. pneumonia*	C. albicans*
25	20.00 ± 0	20.00 ± 0.3	19.66 ± 0.3	20.66 ± 0.3	21.00 ± 0.3	23.66 ± 0	22.00 ± 0.3	18.66 ± 0.3
12.5	18.00 ± 0.3	18.5 ± 0.3	17.00 ± 0	18.66 ± 0.3	19.00 ± 0.3	22.66 ± 0	19.00 ± 0.3	17.66 ± 0.3
6.25	16.66 ± 0.3	16.33 ± 0.3	15.66 ± 0.3	17.33 ± 0.3	16.50 ± 0.5	17.33 ± 0.4	17.50 ± 0.3	15.00 ± 0
3.125	14.33 ± 0.3	14.33 ± 0.3	13.66 ± 0.3	15.33 ± 0.3	14.50 ± 0.5	18.33 ± 0.4	15.00 ± 0.3	13.00 ± 0.3
1.56	11.66 ± 0.3	11.66 ± 0.3	11.66 ± 0.3	14.00 ± 0.5	12.00 ± 0.5	15.66 ± 0	13.00 ± 0	12.50 ± 0.3
0.78	9.66 ± 0.3	9.66 ± 0.3	8.66 ± 0.3	12.33 ± 0.3	10.50 ± 0.3	13.00 ± 0.3	10.50 ± 0	9.66 ± 0.3
0.39	7.33 ± 0.3	7.33 ± 0.3	6.33 ± 0.3	10.33 ± 0.3	8.50 ± 0	10.00 ± 0	8.50 ± 0.3	6.66 ± 0.3
AgNO_3_	5.00 ± 0.3	5.00 ± 0.3	4.00 ± 0.3	6.00 ± 0.5	5.00 ± 0.3	6.00 ± 0.3	5.00 ± 0	5.00 ± 0.5
*S. roseolus Supernatant*	0	0	0	0	0	0	0	0

Data are average d of 3 replicates ± SD (standard deviation). One-way ANOVA showed that the *P* value < 0.05, suggesting significant effect of nano-silver on different microorganisms, Tukey HSD* test showed that the only significant variance was between *E. coli* and *C. albicans* with *P* value < 0.05

### Quantitative determination of MIC, MBC, and tolerance level

The MIC and MBC of biosynthesized Ag-NPs were determined as an estimation of their antimicrobial activity against the Gram-positive *L. monocytogenes* and Gram-negative *K. pneumonia* as multidrug-resistant pathogens. Table 2 shows that the MIC values for *L. monocytogenes* were 195 and 97 μg/mL using agar dilution and broth macro-dilution methods, respectively. The MBC values obtained by these two methods were very applicable at a concentration of 195 µg/mL for the same strain. However, lower MIC values of Ag-NPs were detected for the inhibition of the Gram-negative *K. pneumonia* at 48 and 24 μg/mL, using agar dilution and broth macro-dilution, respectively. Results also indicated that the Gram-negative *K. pneumonia* was more sensitive to Ag-NPs than the Gram-positive *L. monocytogenes*. Results in Table 2 show that the broth macro-dilution method is more sensitive than other screening agar methods, thus it is more suitable for rapid quantitative determination of the antimicrobial activity of Ag-NPs. The tolerance level of pathogens towards Ag-NPs was calculated from their respective MIC and MBC values (Table 2). The tolerance level was 2 using broth macro-dilution method, for *L. monocytogenes*, and *K. pneumonia*. In the current study, both MIC and MBC determined using the two dilution methods did not observe any visible growth when plated on solid media after 24 h of incubation, and the MBC was equal to or within 1 or 2 double its MIC.

**Table 2:** The antibacterial activity of biosynthesized Ag-NPs determined as MIC and MBC using agar dilution and broth macro-dilution methods

Concentration (μg/mL)	Listeria monocytogenes		Klebsiella pneumonia	
	Agar dilution	Broth macro-dilution	Agar dilution	Broth macro-dilution
MIC	195	97	48	24
MBC	195	195	97	48
Tolerance level (MBC/MIC)	1	2	2	2

MBC/MIC ratio ≥ 16, the antimicrobial agent is considered bacteriostatic, and MBC/MIC ratio ≤ 4, the agent is considered bactericidal

### SEM imaging

**Fig. 6:**
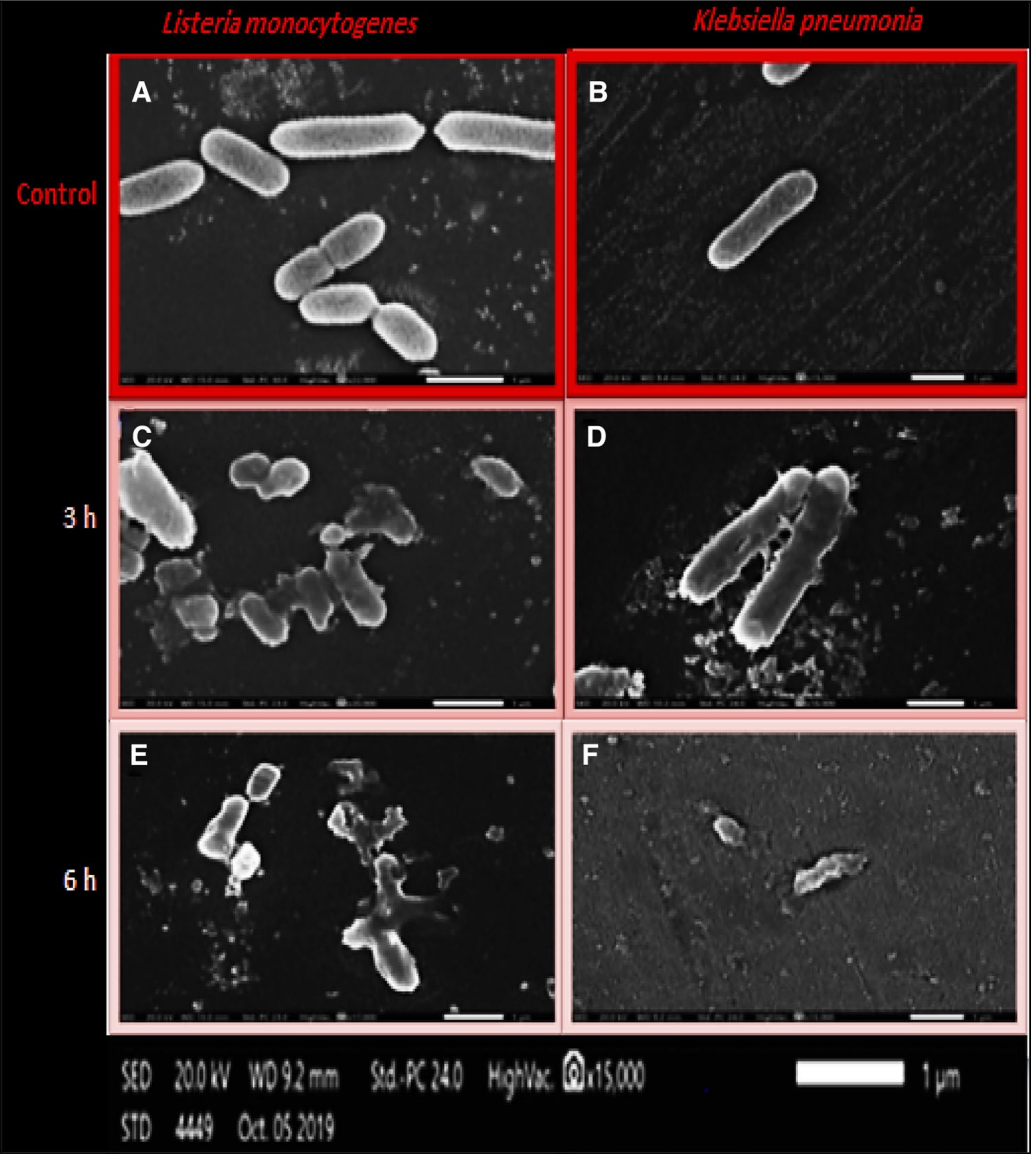
SEM micrograph showing the antimicrobial action of biosynthesized Ag-NPs against *L. monocytogenes* (**A**), and *K. pneumonia* (**B**) at MIC concentration after 3 h (**C**, **D**) and 6 h of incubation (**E**, **F**)

Cells treated with their MIC of biosynthesized Ag-NPs after 3 and 6 h of incubation showed notable morphological alterations as compared to the control with no treatment (Fig. 6). After 3 h of exposure to MIC concentrations, the treated *L. monocytogenes* and *K. pneumonia* cells were tiny, shrunk, diminutive, and dehydrated, with many pits in their surface. Some cells showed large leakage, others appeared misshapen and fragmented. After 6 h of exposure to Ag-NPs, the microbial cells were fully lysed, and the interior dehydrated cellular components and debris were the only observable matrix.

### Cytotoxicity and genotoxicity assessment

**Fig. 7:**
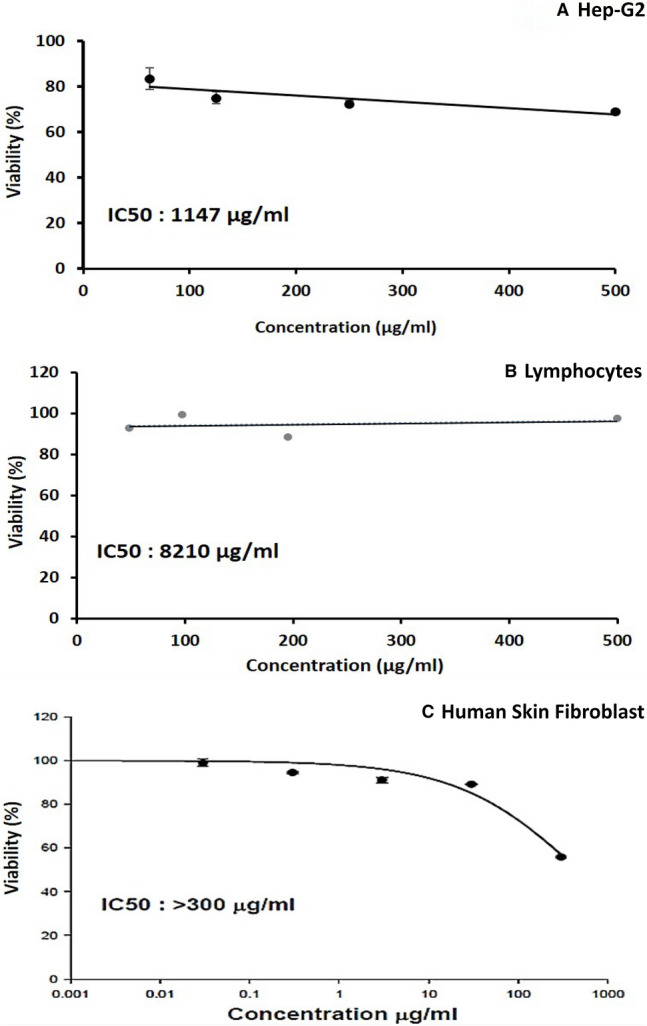
Cytotoxic effect of biosynthesized Ag-NPs against Hep-G2 (**A**), lymphocytes (**B**) using MTT assay (n = 3), and HSF (**C**), using SRB assay, data expressed as the mean value of cell viability (% of control) ± S.D. IC_50_ was calculated to be 1147 µg/mL, 8210 μg/mL, and < 300 μg/mL, respectively

The cytotoxicity of tested samples was measured against Peripheral Blood Lymphocytes normal cells and Hep-G2 malignant cell lines using the MTT assay, as well as against Human Skin Fibroblast (HSF) normal cell lines using the SRB assay. Analysis of cytotoxicity by MTT assay assured the non-cytotoxic impact of biosynthesized Ag-NPs on normal tested cells (Fig. 7 and Table 3). Results revealed that the biosynthesized Ag-NPs have no cytotoxic effect on different cell lines in the tested range (48–500 μg/mL). The cytotoxic effect of biosynthesized Ag-NPs showed IC_50_ of 1147 μg/mL for HepG-2, 8210 μg/mL for normal Peripheral Blood Lymphocytes, and < 300 μg/mL for HSF normal cell lines. The biosynthesized Ag-NPs have no harmful effect on the cell viability of normal Blood Lymphocytes which was almost the same (< 90% viability) for all tested concentrations. Overall, the biosynthesized Ag-NPs (Fig. 7 and Table 3) had significantly higher IC_50_ than its MBC values (97–195 µg/mL) against the two studied multidrug-resistant pathogens (*K. pneumonia* and *L. monocytogenes*).

**Table 3:** Mean value of cell viability of normal and HepG cell-lines (% of control) ± S.D at different concentrations of biosynthesized Ag-NPs. IC_50_ was calculated in µg/mL

Cell line	Concentration	Viability mean	Standard deviation	IC_50_ (μg/mL)
Human skin fibroblast	Control	100	0	
	0.03	98.90	1.69	
	0.3	94.52	0.48	> 300
	3	91.12	1.24	
	30	89.13	0.054	
	300	55.85	0.42	
Hepatocellular carcinoma	500	69.16	0.63	
	250	72.59	1.25	1147
	125	75.15	2.73	
	62.5	83.60	4.75	
Peripheral blood lymphocytes	500	97.64	2.6	
	195	88.54	14.5	8210
	97.5	99.57	1.54	
	48	92.95	8.9	

**Fig. 8:**
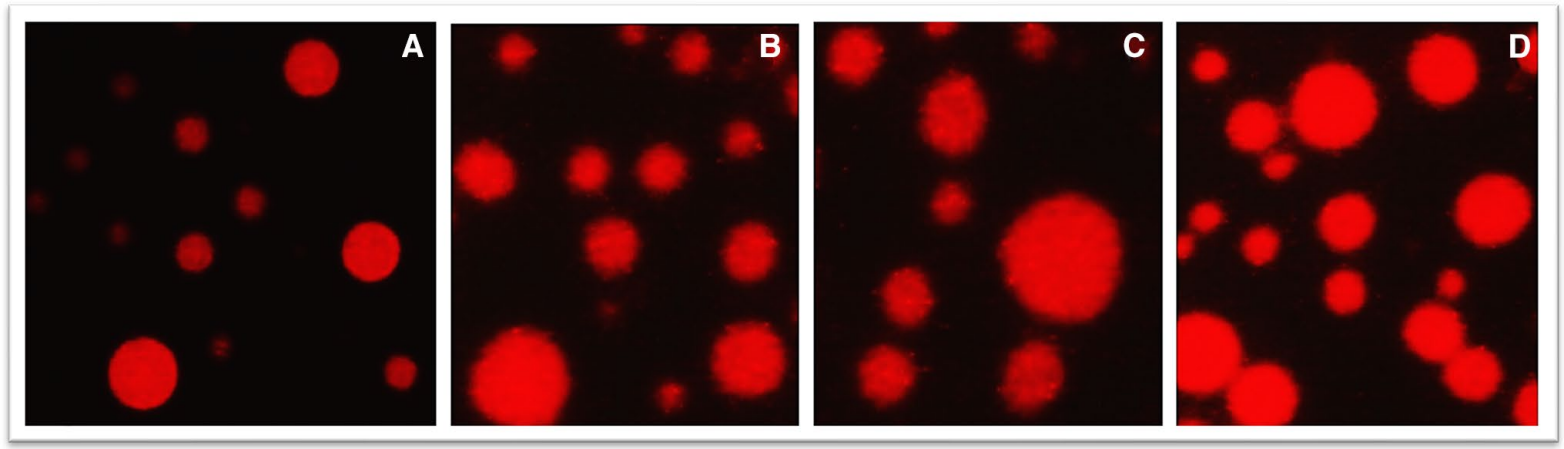
Single cell gel electrophoresis comet analysis of untreated (**A**) and peripheral Blood Lymphocytes (**B**, **C**, **D**) treated with different concentration of biosynthesized Ag-NPs

Comet assay was performed on treated peripheral Blood Lymphocytes to evaluate the genotoxic potential of biosynthesized Ag-NPs. Results showed no fragmentation of DNA and no tail formation for all tested concentrations using Comet electrophoresis (Fig. 8). This is the first report for the genotoxicity assessment of biosynthesized Ag-NPs on peripheral Blood Lymphocytes. No genotoxic was recorded in all treated samples and data obtained (Fig. 8) showed that tail migration is zero and head intensity is 100%.

## Discussion

### Biosynthesis of Ag-NPs, and molecular identification of the selected isolate

In this study, Ag-NPs were successfully synthesized extracellularly within 6 h only, using the culture supernatant of *Streptomyces* strain newly isolated from the Egyptian soil. The biological synthesis of Ag-NPs has received great attention owing to their eco-friendly and cost-effective process. The surface of Ag-NPs is coated with different biomolecules from the *Streptomyces* supernatant making them biocompatible and more safe. Therefore, biological synthesis can offer interesting applications in biomedicine and other related fields (Sharma et al. 2019). The selected isolate was molecularly identified using 16S rRNA gene sequencing. The NCBI database and phylogenetic tree showed 98.74% similarity with *Streptomyces roseolus* strain (NBRC 12,816), with a high genetic relationship confirming its identity as *Streptomyces roseolus* (Fig. 1).

### Characterization of biomediated Ag-NPs

The strong and broad surface-plasmon-resonance (SPR) band that was observed at 470 nm, is characteristic for Ag-NPs (Fig. 2). The intensity of SPR peaks detected by UV–Vis spectroscopy was increased probably due to the increase in Ag-NPs concentration (Ibrahim 2015). Similarly, Alsamhary (2020) reported that Ag-NPs were biosynthesized using *B. subtilis* with a strong broad peak between 400 and 470 nm indicating the formation of Ag-NPs. XRD pattern showed characteristic XRD diffraction peaks as indexed planes of cubic face-centered silver. XRD spectrum was compared with the standard published by the Joint Committee on Powder Diffraction Standards (file no. 04-0783), confirming the crystalline nature of produced Ag-NPs. The peaks intensity reflects the high degree of Ag-NPs crystallinity (Fig. 2). Similarly, Bakhtiari-Sardari et al. (2020) synthesized Ag-NPs using *Streptomyces* sp. OSIP1 with diffraction peaks typical to face-centered cubic silver at 38.2 (111), 44.4 (200), 64.6 (220), and 77.5 (311) corresponding to the crystalline planes of Ag. DLS measures the hydrodynamic diameter of NPs, which is higher than its actual diameter obtained from TEM images, and relating the number % with its PDI, it is suggested that the produced Ag-NPs were highly dispersive (Fig. 3). FTIR analysis (Fig. 4) verified the presence of different functional groups on the surface of biosynthesized Ag-NPs due to the biomolecules in the *Streptomyces* supernatant, that act as reducing and stabilizing agent for the produced Ag-NPs (Zhang et al. 2016). Specific strong absorption bands at 3420 cm^−1^ (N–H stretch) were characteristic of the amine group (Mourdikoudis et al. 2018). Strong band at 2952 cm^−1^ was assigned to aldehydic C–H stretching (Sharma et al. 2019) and bands at 1614 cm^−1^ and 1430 cm^−1^ were assigned to the absorption peaks of (C=C) alkyl and (–C–H) alkanes, respectively (Mourdikoudis et al. 2018). Different absorbance peaks at 1370, 1154, and 1062 cm^−1^ were related to CH_2_ of alkanes, C–O of carboxylic acid, and (C–N) stretching, respectively (Ghaseminezhad et al. 2012; Ibrahim 2015). The amide and aromatic group residues of proteins have a stronger ability to bind metals and form a surface layer on metallic Ag-NPs confirming that proteins act as a capping agent for stabilization (Malarkodi et al. 2013).

### Antimicrobial activity of Ag-NPs, tolerance level, and SEM imaging

Chemical antimicrobial agents are limited to use because various microorganisms have established resistance properties over generations. Ag-NPs have a significant advantage as compared to conventional chemical antimicrobial agents towards the multidrug-resistance problem, and this is because bacteria are less probable to develop resistance to metallic NPs as compared to conventional antibiotics (Loo et al. 2018). The highest antimicrobial activity was against Gram-negative (Table 1 and Fig. 5) probably due to the cell wall structure (Erjaee et al. 2017). Similarly, Huq and Akter (2021) reported that the extracellularly biosynthesized Ag-NPs by *Paenarthrobacter nicotinovorans* MAHUQ-43 exhibited higher antibacterial activity against the Gram-negative *P. aeruginosa* (24.7 mm) than Gram-positive *B. cereus* (19.3 mm).

The tolerance level should be evaluated to determine if the biosynthesized Ag-NPs have a bactericidal or bacteriostatic effect against tested pathogenic strains. When MBC/MIC ratio is ≥ 16, the antimicrobial agent is considered bacteriostatic, and when the ratio is ≤ 4, the agent is considered bactericidal (Das et al. 2017). The National Clinical Committee for Laboratory Standards reported that an agent is considered bactericidal when it causes 99.9% reduction in CFU/mL after 18–24 h of incubation in broth media, however, the microbe is considered as tolerant if its MBC is higher than its MIC by 32-fold or more (Woods and Washington 1995). The tolerance level of 2, for *L. monocytogenes*, and *K. pneumonia* confirmed that biosynthesized Ag-NPs have a biocidal effect against both pathogens (Table 2). Treated-*L. monocytogenes* and *K. pneumonia* with Ag-NPs (Fig. 6) showed notable morphological alterations as compared to the control suggesting the bactericidal effect of biosynthesized Ag-NPs (Sondi and Salopek-Sondi 2004). Likewise, Sondi and Salopek-Sondi (2004) reported that the antimicrobial activity of Ag-NPs against *E. coli*, is probably due to pits formation in the cell wall of treated-pathogen, leading to its death. The peptidoglycan protein layer in bacterial cell wall is negatively charged making more positively charged Ag^+^ ions get stuck in Gram-positive than in Gram-negative. The thicker cell walls of Gram-positive slows down the passage of Ag-NPs and thus, allows fewer Ag-NPs to reach the cytoplasmic membrane. As a result, Gram-negative bacteria are more susceptible than Gram-positive (Ahmed et al. 2016; Erjaee et al. 2017). In addition, the uptake, accumulation, and translocation of Ag-NPs inside the cells depend on the structure of cells, their permeability, and also the size of NPs (Li et al. 2015). Tripathi et al. (2017) reported that Ag^+^ ions can accumulate in the cytoplasm or near cell wall, disrupting the membrane permeability, and facilitating the entrance of Ag-NPs which inhibits the respiratory enzyme(s), and allows the production of reactive oxygen species (Das et al. 2017). Furthermore, Ag-NPs bind to thiol groups (-SH) of enzymes, causing deactivation of enzymes (Ahmed et al. 2016). Moreover, Shahverdi et al. (2007) proposed that Ag^+^ ions can enter the cell and disrupt the hydrogen bonding between DNA strands, denaturing it, so the DNA loses its replication capability and expression of ribosomal proteins.

### Cytotoxicity and genotoxicity assessment

Cytotoxicity is an important factor that affects the safety applications of Ag-NPs (Fig. 7). Dermal contact is one of the most common ways of NPs exposure (Schneider et al. 2009). Therefore, NPs can be used in cosmetics if only their effect and absorption were evaluated on the skin (Kong et al. 2011). So, the use of standard HSF cell lines would be effective to screen in vitro cytotoxicity of the produced Ag-NPs (Fig. 7). The lower toxicity observed on normal cell lines is probably due to the biomolecules present in the biological source used for biosynthesis and capping of Ag-NPs, which influenced its properties (Roy et al. 2019). The potential cytotoxicity of NPs depends on the routes of administration and NPs characteristics, such as size, shape, concentration, and time exposure as well as the capping agent used for stabilization (Niska et al. 2016; Tayel et al. 2017). The effect of different capping agents on cytotoxicity was investigated by Niska et al. (2016) using spherical-shaped Ag-NPs capped with lipoic acid (AgNPs-LA), polyethylene glycol (AgNPs-PEG), or tannic acid (AgNPs-TA), and compared with uncapped ones against human gingival fibroblast cells using MTT assay. Capped-AgNPs-LA had the lowest cytotoxicity, followed by AgNPs-PEG, and AgNPs-TA. This difference in cytotoxicity clearly reveal the influence of capping agents on Ag-NPs cytotoxicity. Furthermore, Paknejadi et al. (2018) reported that chemically-prepared Ag-NPs exhibited higher cytotoxicity on normal HSF cell lines with IC_50_ values of 30.64 μg/mL, after 24 h of incubation. Similarly, Kummara et al. (2016) biosynthesized AgNPs using *Azadirachta indica* leaves extract and compared its cytotoxic effect against HDF cell lines. The biosynthesized AgNPs treatment at different concentrations (0–240 μg/mL) didn't decrease the viability of HDF cells even at higher concentrations, while, chemically synthesized Ag-NPs showed significant toxicity confirming that biosynthesized AgNPs were much safer on normal HDF than chemically synthesized ones. However, the IC_50_ of biosynthesized AgNPs was 60 μg/mL on HDF cell lines (Kummara et al. 2016) which is lower than the biosynthesized one in the current study.

Genotoxicity using Comet assay provides new insight for complete risk assessment of Ag-NPs (Narciso et al. 2020). One of the major issues in the identification of Ag-NPs hazards is its genotoxic potential which is identified by the European Food Safety Authority (EFSA) as an important step in safety evaluation (EFSA Scientific Committee et al. 2016). Genotoxicity data provides a more comprehensive risk assessment of Ag-NPs (Fig. 8). The Comet assay is often used in nano-genotoxicology to identify substances that can cause DNA damage (Glei et al. 2016). The Comet assay is a sharp, easy, and flexible assay used for the identification of DNA degradation in eukaryotic cells. It enables the detection of cross-links, and disruption of double-stranded DNA (Sharma et al. 2021). Similarly, Narciso et al. (2020) reported no genotoxic damage by Comet assay in mice tissues (blood, spleen, liver, kidney, and duodenum) when exposed orally to Ag-NPs of 50–300 mg/kg.wt/day for 3 days. In contrast, Patlolla et al. (2015) reported a significant increase in the genotoxic effects, such as chromosome aberrations, and damaged DNA in Ag-NPs-treated rats for 5 days, at doses of 5–100 mg/Kg.wt/day. Collectively, different physicochemical characteristics, including size, shape, and administration route can lead to different biological activities, thus affecting the outcome of hazard identification (Tayel et al. 2017; Narciso et al. 2020). Overall, the biosynthesized Ag-NPs in this study had significantly higher IC_50_ than its MBC values against the two studied multidrug-resistant pathogens (*K. pneumonia* and *L. monocytogenes*) with no detected genotoxicity. Consequently, it is safer to be used as an effective safe antimicrobial agent at its biocidal concentration.

## Conclusion

*Streptomyces roseolus* was isolated from the Egyptian soil and molecularly identified using 16S rRNA gene sequencing. *S. roseolus* was efficiently used for the first time as a simple, green, cost-effective new bio-reducing agent for extracellular biosynthesis of Ag-NPs, after 6 h only at room temperature. The produced Ag-NPs was characterized using UV–Vis spectroscopy, TEM, DLS, FTIR, and XRD. FTIR analysis confirmed the bio-reduction of Ag^+^ ions to Ag-NPs and its stabilization by different biomolecules in *S. roseolus* supernatant, which probably influenced its biosafety. The MBC of the biosynthesized Ag-NPs against *L. monocytogenes* and *K. pneumonia* were 195 and 48 µg/mL, respectively, with a tolerance level of 2 confirming its biocidal effect. SEM imaging showed destruction of treated pathogen cells after 6 h of exposure to MIC. Biosynthesized Ag-NPs showed IC_50_ of 8210 μg/mL for normal Blood Lymphocytes, and < 300 μg/mL for Human Skin Fibroblast cell lines, which is way higher than other published reports, assuring its safe application as antiseptic and in dermal practice. In the current study, no genotoxicity was recorded for the produced biogenic nano-silver on peripheral Blood Lymphocytes using Comet assay, with zero tail migration and 100% head intensity. The biosynthesized Ag-NPs exhibited strong antimicrobial activity, with no harmful effect on different normal cell lines, indicating their safe usage at their biocidal concentration in many industrial applications.
